# Outcomes of Non-metastatic Colon Cancer: A Single-Center Experience

**DOI:** 10.7759/cureus.17657

**Published:** 2021-09-01

**Authors:** Abdulaziz M Saleem, Wafa Saber, Rawan A Alnajashi, Ebtihal A Alamoudi, Yumn H Shilli, Amani M Aljabarti, Marwan Al-Hajeili

**Affiliations:** 1 Surgery, King Abdulaziz University Faculty of Medicine, Jeddah, SAU; 2 Internal Medicine, King Faisal Specialist Hospital and Research Centre, Jeddah, SAU; 3 Internal Medicine, King Abdulaziz Medical City, Jeddah, SAU; 4 Radiology, King Abdullah Medical City, Jeddah, SAU; 5 Medicine, King Abdulaziz University Faculty of Medicine, Jeddah, SAU

**Keywords:** colon cancer recurrence, non metastatic colo-rectal, colon disease, colo rectal cancer, cancer-specific outcome

## Abstract

Background

Colorectal cancer (CRC) is the most common gastrointestinal cancer. In the Saudi Cancer Registry, CRC ranked as the most common cancer in men and the third most common cancer in women. Data regarding the stage of CRC at presentation and patient demographics and outcomes in Saudi Arabia are lacking. This study aimed to investigate the prevalence, survival, and mortality rates of patients with non-metastatic CRC in a tertiary care hospital in Saudi Arabia.

Methods

We conducted a retrospective chart review of patients diagnosed with adenocarcinoma of the colon or rectum at King Abdulaziz University Hospital between 2013 and 2017. Patients aged ≥18 years who presented with non-metastatic CRC and underwent curative resection were included. Patients with rectal cancer or metastatic colon cancer were excluded. Data on demographic characteristics, histopathological findings, tumor-node-metastasis stage, biomarkers, and surgical interventions were collected. Recurrence-free survival was defined as the time from surgery to the date of recurrence or death. All statistical analyses were performed using Stata/IC 15.1 (StataCorp, College Station, TX, USA).

Results

Among 260 patients diagnosed with CRC, 82 were included based on the inclusion/exclusion criteria. Among those patients, 65.9% were men and 47.5% were Saudi citizens. The mean age at the time of diagnosis was 60.8 years. Fifty-three patients (64.6%) had left-sided colon cancer. The mean tumor diameter was 52.6 mm. Most colon tumors were T3 lesions (71.3%), and 41% of patients did not have lymph node involvement (N0). Most patients (85.1%) underwent open surgery. In the multivariate analysis, only resection margin status and N stage (hazard ratio: 17.7 and 3.7, respectively) were identified as statistically significant factors affecting the recurrence-free survival. The one-, two-, and five-year recurrence-free rates were 80.5%, 66.5%, and 57.1%, respectively, and the one-, two-, and five-year and overall survival rates were 90.3%, 82.5%, and 82.5%, respectively.

Conclusions

We showed significant reductions in recurrence-free and overall survival within the first two years after surgical resection. Further prospective studies are needed to explore predictors.

## Introduction

In 2018, cancer was the second leading cause of death worldwide, with colorectal cancer (CRC) being the third most common cancer in both sexes and the second leading cause of cancer-related deaths worldwide [1]. In Saudi Arabia, CRC is the second most common cancer in both sexes combined, ranking as the most common cancer in men and the third most common cancer in women [2].

Survival of CRC patients is influenced by disease stage at diagnosis [3,4]. The five-year survival rate of patients in whom CRC is detected at a localized stage is as high as 90%. The rate reduces to 70.4% for patients with regional involvement and further reduces to 12.5% for patients with distant metastases [4,5]. In general, the earlier CRC is detected, the greater the chance of survival. A previous study examined mortality rates in 29 selected countries and reported that CRC mortality rates declined in several economically developed regions, including the United States of America, New Zealand, Australia, Western and Eastern Europe, Japan, and South Africa. Screening, earlier diagnosis, lifestyle changes, and advances in treatment have played major roles in improving mortality rates of CRC patients [6-8].

Epidemiological data differ over time and between diverse geographical areas as a result of variable exposure to risk factors, the application of preventive strategies, and the evolution of treatments [9]. This study aimed to investigate the prevalence, survival, and mortality rates of patients with non-metastatic CRC in a tertiary care hospital in Saudi Arabia.

## Materials and methods

Ethical approval

The study design was approved by the Institutional Review Board of King Abdulaziz University, Jeddah, Saudi Arabia.

Study design

We conducted a single-center retrospective chart review of patients diagnosed with histopathologically confirmed adenocarcinoma of the colon or rectum at King Abdulaziz University Hospital over a five-year period from 2013 to 2017.

Participants

Patients aged ≥18 years who presented with non-metastatic CRC and underwent curative resection of the primary tumor were included. Only patients with histologically confirmed adenocarcinoma were included. Patients with radiological or pathological evidence of metastasis or missing clinical or histopathological data were excluded. Patients diagnosed with rectal cancer or appendiceal neoplasms were also excluded.

Classification of primary tumor location

Primary tumors of the cecum, ascending colon, hepatic flexure, and transverse colon were classified as right-sided tumors, while primary tumors of the splenic flexure, descending colon, and sigmoid colon were classified as left-sided tumors.

Data collection

Demographic data included age, sex, and nationality. The relevant histopathological information included the date of diagnosis, tumor location, histological type, tumor diameter, and tumor grade. Well-differentiated tumors were classified as low grade, while poorly or undifferentiated tumors were classified as high grade. Additional data collected included surgical resection margin status, the presence of lymphovascular invasion or perineural invasion, microsatellite instability, and the presence of tumor perforation. 

We collected data on several biomarkers, including KRAS, NRAS, and BRAF mutations, and carcinoembryonic antigen levels. We also collected data on surgical interventions, including the date of surgery, surgical technique (open vs. laparoscopy), number of harvested lymph nodes, and number of involved lymph nodes, as well as the date of last follow-up, presence of recurrence, and date of death (if applicable). Strict anonymity and confidentiality were maintained.

Statistical analysis

The data were checked for missing values before analysis. For descriptive statistics, categorical variables were expressed as numbers and percentages. Continuous variables were expressed as means and 95% confidence intervals (CIs). Recurrence-free survival and overall survival were calculated using the Kaplan-Meier method from the date of surgery to the date of the event. Univariate and multivariate analyses were performed using the Cox proportional hazards model. All statistical analyses were conducted using Stata/IC 15.1 for Mac (StataCorp, College Station, TX, USA). A p-value <0.05 was considered statistically significant.

## Results

Patient characteristics

The patient characteristics are summarized in Table 1. Among 260 patients diagnosed with CRC, 82 were included based on the inclusion/exclusion criteria. Fifty-four patients (65.9%) were men. The remaining 28 patients (34.1%) were women. Less than half of the patients were Saudi citizens (n=39, 47.5%), 19 (23.2%) were Yemeni citizens, and 24 (29.3%) were from other nationalities. The mean age at the time of diagnosis was 60.8 years (95% CI: 57.94-63.69). Fifty-three patients (64.6%) had left-sided colon cancer. The remaining 29 patients (35.4%) had right-sided colon cancer. The majority of the tumors (n=76, 93.8%) were low grade. Only three tumors (3.7%) were high grade. The mean tumor diameter at the time of surgery was 52.6 mm (95% CI: 46.38-58.77). The pathological T stage was pT3 in 57 patients (71.3%). Forty-six patients (60.0%) had lymph node involvement. Sixteen patients (19.5%) had lymphovascular invasion, and 10 patients (12.2%) had perineural invasion. Laparotomy was the most common surgical procedure (n=63, 76.8%), followed by laparoscopy in nine patients (11.0%), other procedures in two (2.4%), and unknown in eight (9.8%). Seventy-four patients (90.2%) had negative resection margins. Tumor perforation was not detected in 78 patients (95.1%). The mean number of harvested lymph nodes was 15.71 (95% CI: 13.75-17.67), while the mean number of involved lymph nodes was 2.33 (95% CI: 1.46-3.19).

**Table 1:** Patient characteristics CEA, carcinoembryonic antigen; SD, standard deviation

Characteristic	Patients (n=82)
Age (years), mean±SD	60.81±1.45
Sex, n (%)	
Male	54 (65.9)
Female	28 (34.1)
Nationality, n (%)	
Saudi	39 (47.5)
Yemeni	19 (23.2)
Other (non-Saudi)	24 (29.3)
Tumor location, n (%)	
Right-sided	29 (35.4)
Left-sided	53 (64.6)
Grade, n (%)	
Low	76 (93.8)
High	3 (3.7)
Undetermined	2 (2.5)
Resection margin, n (%)	
Positive	8 (9.8)
Negative	74 (90.2)
Lymphovascular involvement, n (%)	
Yes	16 (19.5)
No	66 (80.5)
Perineural involvement, n (%)	
Yes	10 (12.2)
No	72 (87.8)
Surgical technique, n (%)	
Laparoscopy	9 (11.0)
Laparotomy	63 (76.8)
Other	2 (2.4)
Unknown	8 (9.8)
CEA level (ng/mL), mean±SD	7.54±1.74
No. of involved lymph nodes, mean±SD	2.33±0.43
No. of resected lymph nodes, mean±SD	15.71±0.98

Study outcomes

The one-, two-, and five-year recurrence-free and overall survival rates were 80.5%, 66.5%, and 57.1% and 90.3%, 82.5%, and 82.5%, respectively. The results of the univariate analysis are shown in Table 2. The hazard ratios (HRs) for female sex, positive resection margin status, and lymphovascular invasion were 0.45 (95% CI: 0.189-1.121, p=0.086), 5.6 (95% CI: 1.95-16.04, p=0.001), and 2.13 (95% CI: 0.89-5.1, p=0.088), respectively. T (HR, 2.57; 95% CI: 1.23-5.34, p=0.011) and N stages (HR, 2.336; 95% CI: 1.41-3.85, p=0.001) were significant factors in determining patient outcomes. Recurrence-free survival was poorer for patients who received chemotherapy compared to patient who did not receive chemotherapy as part of their treatment process (HR, 2.29; 95% CI: 1.02-5.16, p=0.045).

**Table 2:** Results of the univariate analysis CEA, carcinoembryonic antigen; CI, confidence interval; HR, hazard ratio; TNM, tumor–node–metastasis *p<0.05; **p<0.01; ***p<0.001

Variable		Univariate analysis		
		HR	95% CI	p-Value
Age (years)		0.996	0.964-1.029	0.804
Sex		0.450	0.180-1.121	0.086
Nationality				
Yemeni to Saudi		2.143	0.886-5.182	0.091
Other (non-Saudi) to Saudi		1.117	0.411-3.032	0.828
Tumor location		1.205	0.794-1.829	0.381
Histological grade				
High to low		0.899	0.121-6.697	0.917
Undetermined to low		3.878	0.896-16.786	0.070
Resection margin		5.606	1.958-16.041	0.001**
Lymphovascular involvement		2.134	0.893-5.100	0.088
Perineural involvement		1.115	0.384-3.240	0.841
Tumor perforation		1.183	0.158-8.855	0.870
Tumor diameter (mm)		1.006	0.993-1.018	0.376
Surgical technique				
Laparoscopy		4.492	1.294-15.597	0.018*
Laparotomy		4.76E+09	6.24E+08-3.63E+10	<0.001***
Other		1.07E+10	–	–
TNM T stage		2.572	1.237-5.348	0.011*
TNM N stage		2.336	1.416-3.855	0.001**
CEA level (ng/mL)		1.021	0.999-1.043	0.059
No. of involved lymph nodes		1.119	1.030-1.216	0.008**
No. of resected lymph nodes		0.994	0.950-1.040	0.794
Chemotherapy		2.296	1.020-5.166	0.045*

Multivariate analysis confirmed positive resection margin status (HR, 17.72; 95% CI: 2.01-155.87, p=0.01) and N stage (HR, 3.73; 95% CI: 1.24-11.16, p=0.019) as independent risk factors for recurrence (Table 3).

**Table 3:** Results of the multivariate analysis CI, confidence interval; HR, hazard ratio; N/A, not available; TNM, tumor–node–metastasis *p<0.05

Variable	Multivariate analysis		
	HR	95% CI	p-Value
Age (years)	1.047	0.992-1.104	0.096
Sex	0.147	0.017-1.275	0.082
Nationality	0.944	0.494-1.802	0.861
Tumor location	1.108	0.465-2.642	0.817
Histological grade	1.432	0.413-4.969	0.572
Resection margin	17.723	2.015-155.874	0.010*
Lymphovascular involvement	0.144	0.018-1.128	0.065
Perineural involvement	2.48E-18	0-N/A	1.000
Tumor perforation	9.04E-18	0-N/A	1.000
Tumor diameter (mm)	0.992	0.958-1.029	0.675
TNM T stage	1.355	0.401-4.577	0.625
TNM N stage	3.730	1.246-11.166	0.019*

## Discussion

CRC is one of the most common malignancies affecting both sexes. CRC ranked third worldwide, and is the second leading cause of cancer-related death [1]. Survival outcomes are influenced by the stage of the disease at diagnosis. The five-year survival rate for Dukes’ stage A disease is >90%, compared to only 5% for Dukes’ stage D disease [10]. Approximately two-thirds of patients present with early-stage disease and are potentially treatable with surgery, chemotherapy, and/or radiotherapy. Nevertheless, 50%-60% of patients develop recurrence with metastatic disease [11]. These findings are consistent with the recurrence-free survival rates obtained in this study (one, two, and five years: 80.5%, 66.5%, and 57.1%, respectively).

The outcomes of CRC patients differ globally. In high-income countries, such as the United States of America and Canada, the five-year survival rate has reached approximately 65%, while in lower-income regions, such as Latin America, Asia, and sub-Saharan Africa, it is <50% [6,12]. The differences in mortality rates may be explained by adherence to up-to-date evidence-based practices and surveillance methods in high-income countries and the lack of these standards in lower-income regions [13]. Other factors, such as lifestyle and cultural beliefs, may explain some of the variation in survival [14,15]. 

Tumor location was not a statistically significant factor for predicting survival in this study. However, it was noted that recurrence-free survival was shorter in patients with right-sided CRC. Differences in survival have been reported between patients with right- and left-sided colon cancer in a study using the Surveillance, Epidemiology, and End Results Program database [16], where patients with right-sided colon cancer had a 5% increased risk of mortality than patients with left-sided colon cancer. Lymph node involvement, which differentiates stage III from stage I/II colon cancer, is a significant prognostic factor in non-metastatic colon cancer [17]. The number of harvested lymph nodes and further pathological evaluation have been reported to affect both the staging accuracy and the tumor outcomes in node-negative, as well as node-positive, patients and is a requisite for adjuvant treatment after curative resection [18,19]. 

A previous study [20], which considers the lymph node ratio (LNR) to be a reliable predictor of the risk of progression, supports these findings. When the LNR was >0.194, the disease-free survival of patients with stage III disease was 45%, and when the LNR was <0.194, the disease-free survival was 71%. In patients with ≥12 harvested lymph nodes, the LNR can help identify with high specificity (86%) those who are less likely to experience disease recurrence (LNR, <0.257).

In this study, we found that lymph node status was a statistically significant factor in determining patient survival (HR, 3.7; p=0.019), which is consistent with the literature. The reason why some patients experience recurrence after curative resection may be explained by inadequate lymphadenectomy, which may result in residual nodal disease [21]. Univariate analysis of 36,712 cases in which half of the identified cases (50.9%) had ≥12 harvested lymph nodes showed an overall improvement in survival with a difference in median survival of 53 vs. 66 months, respectively (p<0.001) [17].

In another study by Le Voyer et al. [19], there was an absolute improvement in five-year survival of 23% with an increasing number of harvested lymph nodes in node-positive patients (40 vs. ≤10 lymph nodes). Similarly, the five-year survival rate increased by 20% when >35 lymph nodes were resected in patients with ≥4 positive lymph nodes. These findings may be attributable to the high-quality pathology service, accurate tumor staging, and adequate surgical intervention [22]. Conversely, the improvement in our study was unrelated to the number of resected lymph nodes. This may be explained by the lack of variation in the number of resected lymph nodes. 

Further studies [19,23-26] have shown that the greater the number of harvested lymph nodes, the more favorable the outcome. The mainstay of treatment for CRC is surgical resection, with an estimated 92% of patients with colon cancer and 84% of patients with rectal cancer undergoing surgical resection as the primary treatment [27,28]. The resection margins after colorectal surgery include the proximal, distal, mesenteric, and circumferential radial margins [28].

In this study, we showed that a positive resection margin was associated with a poorer survival (HR, 17.7; p=0.01). This finding is consistent with the findings of a study by Andreou et al. [29] who reported that overall survival after surgical resection was influenced by resection margin status.

This study had several limitations. First, the sample size was limited. This may have led to the failure in identifying factors that determine the outcomes of patients with CRC. Second, laparoscopy has only recently been introduced and, due to the small sample size, no significant differences were found between the laparoscopy and open surgery groups.

## Conclusions

We showed significant reductions in recurrence-free and overall survival within the first two years after surgical resection. In our study, we reported that positive margins and N staging were independent variables to affect patient outcome following curative resection. Our results are similar to those published previously, although the magnitude is higher. Most of our patients underwent laparotomy because of the small sample size, and we could not calculate the HR for this variable. Further prospective studies are warranted to further investigate predictors of survival outcomes in patients with CRC.
